# Cell entry of a host-targeting protein of oomycetes requires gp96

**DOI:** 10.1038/s41467-018-04796-3

**Published:** 2018-06-14

**Authors:** Franziska Trusch, Lars Loebach, Stephan Wawra, Elaine Durward, Andreas Wuensch, Nurul Aqilah Iberahim, Irene de Bruijn, Kevin MacKenzie, Ariane Willems, Aleksandra Toloczko, Javier Diéguez-Uribeondo, Tim Rasmussen, Thomas Schrader, Peter Bayer, Chris J. Secombes, Pieter van West

**Affiliations:** 1 Aberdeen Oomycete Laboratory, Institute of Medical Sciences, University of Aberdeen, Aberdeen, AB25 2ZD Scotland UK; 2 International Centre for Aquaculture Research and Development (ICARD), University of Aberdeen, Aberdeen, AB25 2ZD Scotland UK; 3 School of Fisheries and Aquaculture Sciences, Universiti Malaysia Terengganu, 21030 Kuala Terengganu, Terengganu Malaysia; 4 Microscopy and Histology Facility, Institute of Medical Sciences, University of Aberdeen, Aberdeen, AB25 2ZD Scotland UK; 5 Department of Mycology, Real Jardín Botánico CSIC, Madrid, 28014 Spain; 6 Institute of Medical Sciences, University of Aberdeen, Aberdeen, AB25 2ZD Scotland UK; 7 Organic Chemistry, University of Duisburg-Essen, Essen, 45117 Germany; 8 Structural and Medicinal Biochemistry, Centre for Medical Biotechnology (ZMB), University of Duisburg-Essen, Essen, 45117 Germany; 9 Scottish Fish Immunology Research Centre, Institute of Biological and Environmental Sciences, University of Aberdeen, Aberdeen, AB24 2TZ Scotland UK; 10 Present Address: Botanical Institute, Genetical Institute, University of Cologne, Cologne, 50674 Germany; 11 Present Address: Netherlands Institute for Ecology (NIOO), Wageningen, 6708 PB Netherlands

## Abstract

The animal-pathogenic oomycete *Saprolegnia parasitica* causes serious losses in aquaculture by infecting and killing freshwater fish. Like plant-pathogenic oomycetes, *S. parasitica* employs similar infection structures and secretes effector proteins that translocate into host cells to manipulate the host. Here, we show that the host-targeting protein SpHtp3 enters fish cells in a pathogen-independent manner. This uptake process is guided by a gp96-like receptor and can be inhibited by supramolecular tweezers. The C-terminus of SpHtp3 (containing the amino acid sequence YKARK), and not the N-terminal RxLR motif, is responsible for the uptake into host cells. Following translocation, SpHtp3 is released from vesicles into the cytoplasm by another host-targeting protein where it degrades nucleic acids. The effector translocation mechanism described here, is potentially also relevant for other pathogen–host interactions as gp96 is found in both animals and plants.

## Abstract (web summary)

The pathogenic oomycete *Saprolegnia parasitica* secretes effector proteins that translocate into host cells through unclear mechanisms. Here, Trusch et al. show that the uptake of effector protein SpHtp3, resulting in RNA degradation, depends on a gp96-like host receptor and a second effector protein.

## Introduction

Oomycetes (or watermolds) are eukaryotic microbes that are among the most devastating pathogens of animals and plants with a huge economic and environmental impact in cultured as well as natural ecosystems^1–4^.

Similar to pathogenic fungi, oomycetes can also secret effector proteins that enter the host to establish an infection. They assist the invasion and propagation of the pathogen by reducing the host resistance and overcoming immune responses as well as adapting the host metabolism to the benefit of the pathogen^3,5^. However, a detailed molecular understanding of the translocation of effector proteins from oomycetes into host cells is missing.

In plant-pathogenic oomycetes from the order Peronosporales, a large group of effector proteins are characterised by an N-terminal RxLR motif (Arg–Xaa–Leu–Arg)^5–8^. Although, the RxLR motif is highly conserved, its precise role in the translocation mechanism of effectors into host cells is under debate^9–13^. It is postulated that the RxLR motif of effectors from *Phytophthora infestans* itself might be involved in the uptake by binding to phospholipids in the host membrane^8^. However, recently it was shown that the RxLR motif of the AVR3a effector from *P. infestans* is cleaved off before it is secreted from the pathogen^13^. Following the sequence homology to the PExEL and TExEL motifs in *Plasmodium falciparum* and *Toxoplasma gondii*, respectively^14,15^, to the conserved RxLR motifs in *P. infestans* and *Phytophthora sojae* could also work as a sorting signal in the pathogen itself^13^, which directs the effectors to the export pathway while the translocation into the host is mediated by a translocon^16^.

Little is known about effector proteins from the fish-pathogenic *Saprolegnia parasitica* beside the pathogen-independent uptake of SpHtp1^11^. SpHtp1 is expressed during early stages of infection and self-translocates into host cells in a pathogen-independent manner by binding to tyrosin-O-sulphates. Here, we characterise another host-targeting protein (SpHtp3) from *S. parasitica* and reveal a model for the translocation mechanism. After secretion by *S. parasitica*, SpHtp3 binds to the surface of the host cell and is taken up via a lipid-raft associated gp96-like receptor. Inside the cell, SpHtp3 is released from its vesicles by another host-targeting protein (SpHtp1). Once in the cytosol, SpHtp3 is able to degrade RNA with its bifunctional nuclease domain. Furthermore, translocation of SpHtp3 into host cells was inhibited with supramolecular tweezers^17,18^ providing a promising tool for uptake inhibition studies of other host-targeting proteins.

## Results

### Infection structures of *S. parasitica*

**Fig. 1:**
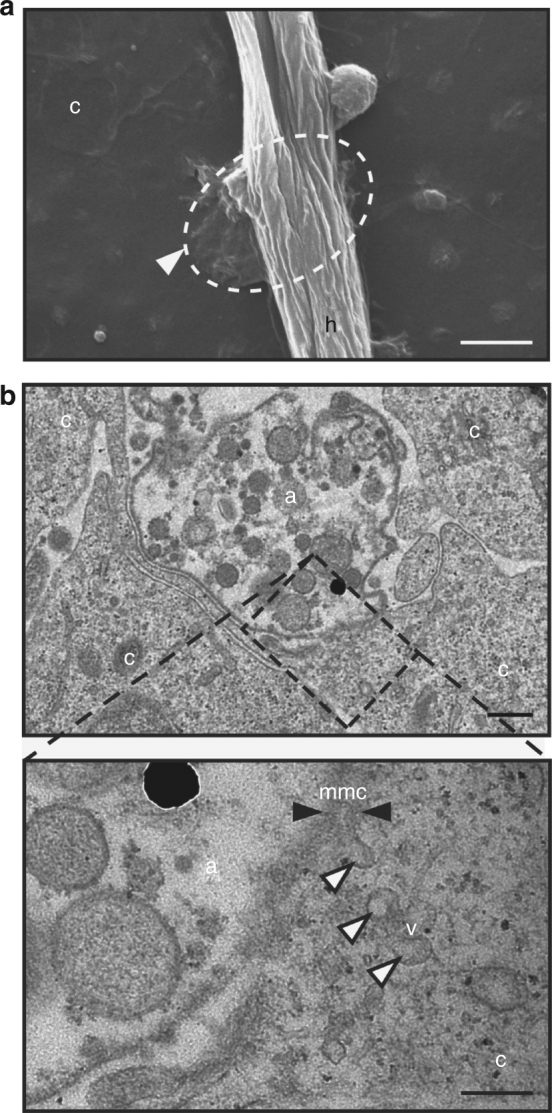
Infection structure of *S. parasitica*. **a** SEM of an hyphae of *S. parasitica* (h) attached to the surface of a fish cell (c). The arrowhead points to an adhesorium-like structure. It is localised underneath the hyphae and fused with the cell membrane. Scale bar: 2 µm. **b** TEM of the adhesorium-like structure (a) at the tip of a *S. parasitica* hyphae with a direct membrane contact (mmc, black arrowheads) with the host cell (c). Magnification of the side of contact (dashed box) reveals expansion and invagination of membranes and numerous vesicles (v, white arrowheads). Scale bars: 0.2 µm

Little is known about how animal-pathogenic oomycetes infect their hosts because most research has been performed with plant-pathogenic oomycetes that form highly specialised infection structures such as appressoria and haustoria^19,20^. Like the plant pathogen *P. infestans*, also the animal pathogen *S. parasitica* forms an infection structure on the surface of fish cells, which resembles an adhesorium rather than a haustorium (Fig. 1a). The adhesorium remains in place until later stages of infection. Indeed, the pathogen and the host membranes are in close proximity with some contacts and a high number of vesicle-like structures are formed (Fig. 1b) allowing for possible exchange of nutrients and effector proteins as has also been suggested for plant-pathogenic oomycetes and fungi^21,22^.

### Pathogen-independent translocation of SpHtp3 into host cells

**Fig. 2:**
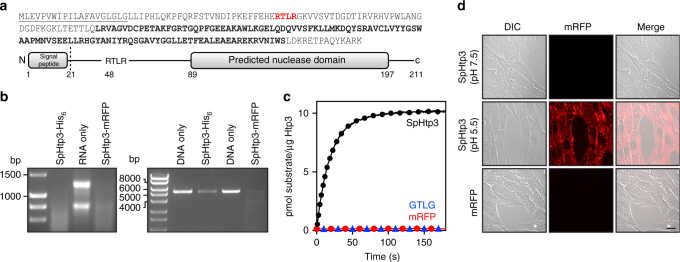
SpHtp3 is a self-translocating nuclease. **a** Amino acid sequence of SpHtp3 (top), including the secretion signal (M1-G21, underlined), the RxLR sequence (R48-R51, red) and the predicted nuclease domain (L89-S197, bold). Protein domain structure of SpHtp3 (bottom). **b** Visualisation of RNA (left, RTG-2 cell RNA) and DNA (right, linearised pET21b) degrading activities of SpHtp3-His_6_ and SpHtp3-mRFP (*n* = 3). **c** Real-time ribonuclease activity assessment of SpHtp3 wt (black) compared to a negative control (SpHtp1-mRFP, red) and a non-functional mutant of SpHtp3 (GTLG, blue) with RNaseAlert® (*n* = 2). **d** Autonomous translocation activity of recombinant SpHtp3-mRFP into living RTG-2 cells at pH 7.5 and 5.5. The control (mRFP only) does not show any translocation. Scale bar: 20 µm (*n* = 3)

Although effector proteins are essential to establish an infection, their pathogen-independent translocation and the exact translocation route into the host are not clear^9–12^. To investigate the translocation process of host-targeting proteins secreted by *S. parasitica*, we have used SpHtp3 (*S. parasitica* host-targeting protein 3) as a model protein since it contains characteristics typical for effector proteins. SpHtp3 comprises a signal peptide for secretion, an RxLR sequence (Arg–Thr–Leu–Arg) and the effector domain is a putative Staphylococcal nuclease domain (SNase, *E* value: 7.3e^−23^, Pfam-A ID: PF00565) (Fig. 2a). In addition, SpHtp_3_-like genes can be found in more than 40 other species being pathogenic to animal and plants (Supplementary Table 1). As expected by the conserved active site, recombinant SpHtp3 shows RNA as well as DNA degradation activity (Fig. 2b) like the *Staphylococcus aureus* nuclease^23^. The specific activity of SpHtp3 was determined by real-time fluorescence imaging to be 30 nmol min^−1^ mg^−1^ (kcat: 0.024 s^−1^), which is also similar to the activity of SNAse (Fig. 2c) and shows a general salt dependency with a clear reduction by Mg^2+^ and SO_4−_ ions (EC_50_ = 0.35 mM for MgSO_4_^23^, Supplementary Fig. 1a and b). RNA degradation by a possible RNase contamination from the purification process could be excluded by control experiments (Supplementary Fig. 1c–e).

In addition, we have used a recombinant protein construct of SpHtp3 fused to mRFP to investigate the translocation ability into living RTG-2 trout fibroblast cells (Fig. 2d and Supplementary Fig. 1f). Indeed, SpHtp3 showed self-translocation into fish cells and is located in vesicle-like structures. However, in contrast to SpHtp1^11^, the uptake of SpHtp3-mRFP into RTG-2 cells is more efficient at a lower pH of 5.5. The pH-dependency of the SpHtp3 uptake could also be observed in other fish cell lines (Supplementary Fig. 1g) but is unlikely to be caused by structural changes considering the highly overlapping CD spectra of SpHtp3 at pH 5.0, 6.0 and 7.0 (Supplementary Fig. 1h).

### SpHtp3 is taken up into host cells via its C-terminus

**Fig. 3:**
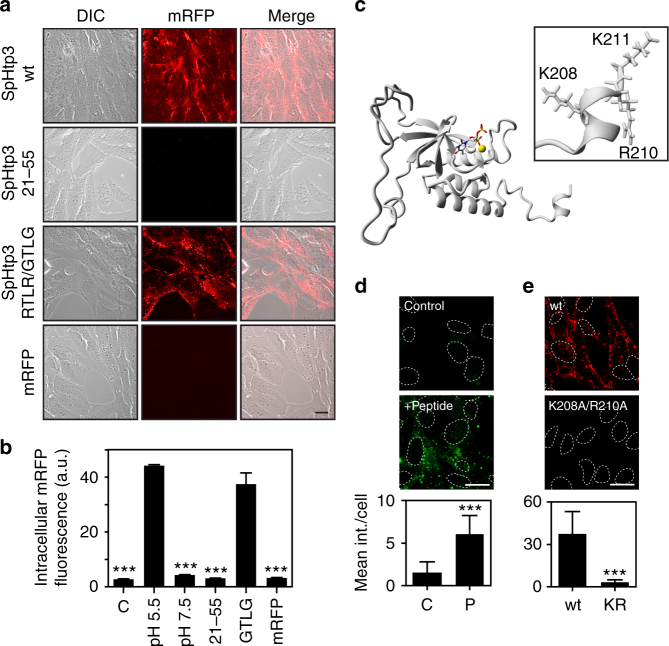
SpHtp3 self-translocates into host cells via its C-terminus. **a** Self-translocation of SpHtp3-mRFP wt, SpHtp3-mRFP_21–55_ (containing the RTLR sequence), a mutant of SpHtp3-mRFP^RTLR/GTLG^ and mRFP only into living RTG-2 cells at pH 5.5. Scale bar: 20 µm (*n* = 3). **b** Quantitative FACS analysis of RTG-2 cells from Figs. 2d and 3a. Error bars denote s.e.m. (*n* = 3). *** *p* < 0.001 (one way ANOVA). **c** Homology model of SpHtp3 calculated by YASARA containing the natural ligands (nucleotide analogue and Ca^2+^ ion (yellow)). The inset shows the structural details of the very C-terminus of SpHtp3 containing a short charged helix built up by an YKARK sequence. **d** Self-translocation of a FITC-labelled C-terminal peptide of SpHtp3 (201–211 aa, P) into RTG-2 cells at pH 5.5. Nuclei are indicated by dashed lines. Error bars denote s.e.m. (cells: 50). ***p < 0.001 (*t*-test). Scale bar: 20 µm (*n* = 3). **e** Self-translocation of a mutant of SpHtp3-mRFP (K208A/R210A, KR) in comparison to SpHtp3-mRFP wt into RTG-2 cells at pH 5.5. Nuclei are indicated by dashed lines. Error bars denote s.e.m. (cells: 50). ***p < 0.001 (*t*-test). Scale bar: 20 µm (*n* = 3)

We have chosen SpHtp3 as a model protein since it also contains an RxLR motif, which is thought to be involved in the translocation of effectors from plant-pathogenic oomycetes^8^. However, the translocation of SpHtp3 appears to be RxLR-independent since a C-terminal truncated version of SpHtp3 (21–55 aa), containing the RxLR motif, does not translocate into RTG-2 cells (Fig. 3a). Whereas, an RxLR mutant (RTLR/GTLG) of the full-length protein still enters the cells (Fig. 3b). Surprisingly, although the mutation does not include active site residues, this mutant is non-functional and lacks RNA degrading activity (Fig. 2c). Since the whole N-terminus up to the core domain was not able to translocate mRFP into cells, we speculated that the uptake motif is localised after the core domain in the C-terminus of SpHtp3. Despite the low sequence similarity between SpHtp3 and SNAse (30% identity; Supplementary Fig. 2a), both share the same core domain structure, while the flexible termini differ (Fig. 3c and Supplementary Fig. 2b–d).

The C-terminus of SpHtp3 (Ala185–Ala189) is predicted to form a short helix with charged amino acids (YKARK) (Fig. 3c and Supplementary Fig. 2e), and it is therefore, a promising structure for ionic interactions between SpHtp3 and the cell membrane. Amongst SpHtp3-like proteins, the C-terminus is remarkably conserved and by sequence alignment, a general binding motif can be proposed (Supplementary Fig. 2f). Indeed, the isolated C-terminus of SpHtp3 (200–211 aa) coupled to FITC localises, like SpHtp3, into vesicular structures (Fig. 3d). Consistently, a mutant of the SpHtp3 full-length protein carrying a double mutation (K208A/R210A) was unable to self-translocate into fish cells (Fig. 3e).

### A gp96-like protein is a host receptor of SpHtp3

**Fig. 4:**
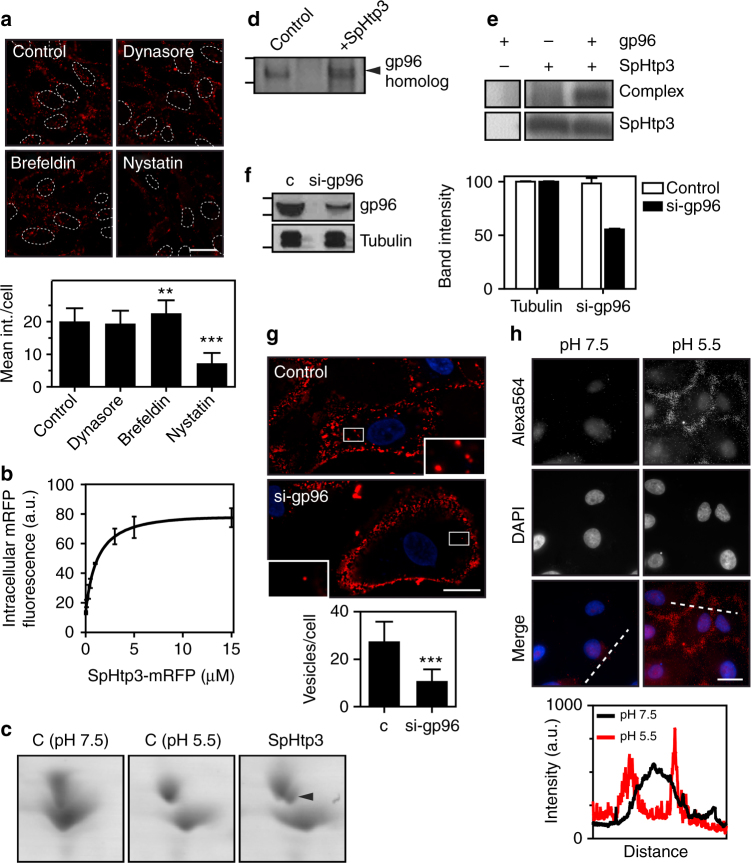
SpHtp3 is taken up via a gp96-like receptor. **a** Uptake inhibition of SpHtp3-mRFP into RTG-2 cells pre-incubated for 1 h with the inhibitors dynasore, brefeldin A or nystatin (top) and respective quantification (bottom). Nuclei are indicated by dashed lines. Error bars denote s.e.m. (cells: 50). ***p < 0.001, **p < 0.01 (one way ANOVA). Scale bar: 20 µm (*n* = 3). **b** FACS analysis of the saturable uptake of SpHtp3-mRFP into RTG-2 cells at higher concentrations. Error bars denote s.e.m. (*n* = 3). **c** Appearance of an additional shifted spot (arrowhead) in 2D-PAGE of a RTG-2 cell lysate for a gp96-like protein after incubation with SpHtp3 compared to control samples (c) at different pH values. **d** Elution fraction of a pull down experiment of an RTG-2 lysate with SpHtp3. An additional band (arrowhead) appears in the pull down with SpHtp3-mRFP, compared to a control with lysate only. LC-MS/MS analysis identified peptides for a gp96-like protein. **e** In vitro complex formation of recombinant SpHtp3-His_6_ with recombinant gp96 (from dog) after cross-link verified by detection by an anti-His antibody. An additional band which only appears in the sample with both proteins is highlighted (complex) (*n* = 3). **f** Knock down of gp96 in A549 cells by siRNA by 50%, left: western blot of control cells and the siRNA-treated sample, right: quantification of bands from the western blot. Error bars denote s.e.m. (*n* = 2). **g** Reduction of gp96 protein results in a reduced uptake of SpHtp3 into A549 cells with fewer vesicles per cell (see magnifications). Error bars denote s.e.m. (cells: 50). ***p < 0.0001 (*t*-test). Scale bar: 20 µm (*n* = 2). **h** Cell surface exposure of gp96 in human A549 cells at different pH as indicated. 2.5 μg α-gp96 antibody was incubated with living cells to exclude intracellular gp96 and to stain surface exposed gp96 only. Pictures were taken with a Zeiss Imager M2. Scale bar: 20 µm. Graph represents the fluorescence intensity of one cell for each pH as indicated by dashed lines (*n* = 2)

To narrow down the cell entry mechanism of SpHtp3, fish cells were treated with different compounds known to inhibit key factors of common cell entry pathways (Fig. 4a). The inhibitor studies support a lipid raft dependent-translocation process for SpHtp3 (nystatin) and excluded clathrin-mediated endocytosis (dynasore). The entry of SpHtp3 into host cells is increased with increasing temperatures, supporting a lipid-mediated process dependent on the fluidity of the cell membrane (Supplementary Fig. 3a). On the other hand, the uptake of SpHtp3 into RTG-2 cells is saturable (Fig. 4b), which excludes a purely diffusion-driven process. In contrast to SpHtp3, another effector protein from *S. parasitica* SpHtp1, expressed during earlier stages of infection^11^, is translocated via a different route (inhibitor of clathrin-mediated endocytosis, Supplementary Fig. 3b). To identify a potential receptor for SpHtp3, we have performed 2D-PAGE analysis of proteins from trout RTG-2 cells incubated with and without SpHtp3 that revealed an additional spot corresponding to a gp96-like protein with 78% identity (GPR94, endoplasmin; GSONMT00046981001) (Fig. 4c). In the next step, we performed a pull down experiment of an RTG-2 cell lysate with SpHtp3 to proof a complex formation (Fig. 4d). An additional band appeared at the size of gp96 and LC-MSMS analysis confirmed the presence of peptides of a gp96-like protein (GSONMT00046981001). The direct interaction between recombinant SpHtp3-His_6_ and recombinant gp96 (no tag) was also confirmed by a cross-link experiment, which revealed another band at the height of a complex (130 kDa) detected by an α-His antibody (Fig. 4e). To investigate the potential role of the gp96-like protein in the translocation of SpHtp3, we have knocked down gp96 in human A549 cells (Fig. 4f) resulting in a clear reduction in intracellular localised SpHtp3-mRFP vesicles (Fig. 4g). Interestingly, and in agreement with the pH-dependency of the uptake of SpHtp3 into fish RTG-2 cells and human A549 lung cells (Fig. 2d and Supplementary Fig. 3c), is the relocalisation of gp96 from the cell centre to the cell membrane in human cells at a lower pH (Fig. 4h). Furthermore, the uptake of SpHtp3 into A549 cells was reduced remarkably by the simultaneous incubation with an α-gp96 antibody compared to a control with SpHtp3 only (24 vs. 2 vesicles per cell on average) (Supplementary Fig. 3d).

### A supramolecular compound inhibits translocation of SpHtp3

**Fig. 5:**
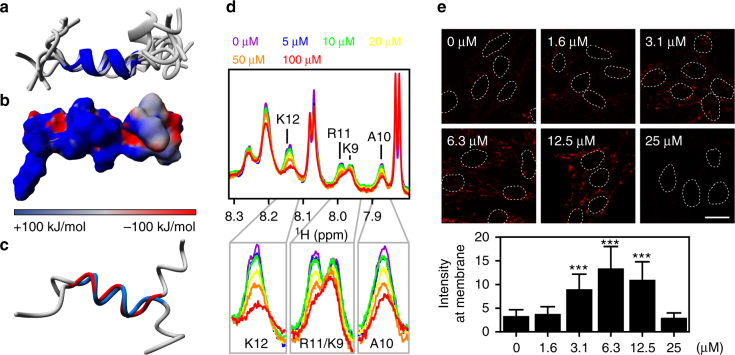
Molecular tweezers inhibit the translocation of SpHtp3. **a** Superimposition of the 10 lowest-energy NMR-based structures of the C-terminal peptide of SpHtp3 with the central helix (P204-K211) highlighted. **b** Electrostatic surface presentation of the C-terminal peptide of SpHtp3. Positive, neutral and negative charges are displayed in blue, grey and red, respectively. **c** Superimposition of the NMR-based structures of the C-terminal peptide wt (blue) and the double mutant (K208A/R210A, red) of SpHtp3 with the central helix (P204-K211). **d** ^1^H-1D NMR titration experiments of the SpHtp3 peptide with a stepwise increasing amount of tweezers as indicated. Decreasing signal intensities indicate an interaction of both. **e** Effect of molecular tweezers on the translocation of SpHtp3-mRFP into RTG-2 cells. With increasing tweezers’ concentrations, the uptake and cell surface binding of SpHtp3 are interrupted. Nuclei are indicated by dashed lines. Error bars denote s.e.m. (cells: 50). ***p < 0.001 (one way ANOVA). Scale bar: 20 µm (*n* = 3)

The C-terminus of SpHtp3 is rich in Lys and Arg, and thereby a promising target for supramolecular hydrogen phosphate tweezers^18^. These tweezers are supramolecular ligands designed to modulate protein–protein interactions by specifically targeting defined areas on a protein in a predictable manner. Hydrogen phosphate tweezers bind selectively Lys and Arg on protein surfaces by drawing the positively charged side chains of Lys and Arg into the tweezers’ tailored cavity. To investigate the interaction between the tweezers and the SpHtp3 translocation module, we have calculated the solution structure of the C-terminus (Fig. 5a). In line with the homology model (Fig. 3c), the peptide forms a short but highly positively charged helix (Fig. 5b). The peptide with the double mutant (K208A/R210A) retains the helical character but with a reduced positive charge due to the replacement of the positively charged Lys/Arg by the hydrophobic Ala (Fig. 5c). We used the structure information for ^1^H-1D titration experiments of the peptide with a stepwise increasing amount of tweezers (1:200–1:4 tweezers:SpHtp3) (Fig. 5d). Since Arg11 and Lys12 show already a loss of signal intensities at low SpHtp3:tweezers ratios (200:1), these residues are expected to be the main binding sites and indicate a high affinity binding constant (*K*_D_s in the lower μM range). Other signals (Lys9 or Ala10 that are also a part of the motif) undergo only weak changes pointing towards a close proximity to the tweezers, while signals for the remaining residues underwent only unspecific changes and are not involved in the peptide–tweezers complex formation.

Next, we tested the potential of the tweezers to block the translocation of SpHtp3 into RTG-2 cells (Fig. 5e). At lower concentrations of the tweezers, only the uptake of SpHtp3 into cells is blocked, which leads to an accumulation at the cell surface. However, with higher tweezers concentrations binding of SpHtp3 to the cell membrane is also interrupted indicating a two-step translocation process of SpHtp3 as was already observed in the α-gp96 blocking experiment (Supplementary Fig. 3d).

**Fig. 6:**
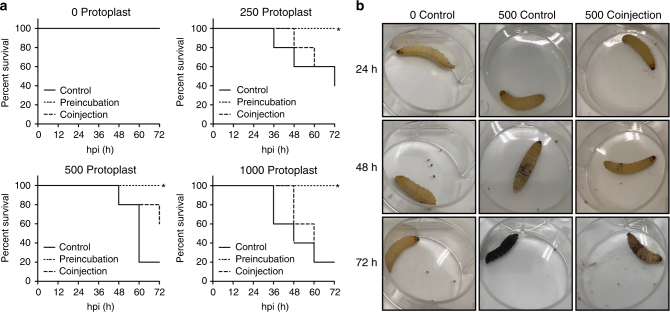
Survival of *G. mellonella* larvae infected with *S. parasitica* after treatment with molecular tweezers. **a** Each group was injected with the amount of protoplasts of *S. parasitica* as indicated (0, 250, 500, 1000). The experiment was performed with five larvae per condition. control: injected with protoplasts only; preincubation: protoplasts were preincubated for 1 h with 5 mM molecular tweezers; coinjection: 5 mM molecular tweezers were injected 1 h before injection of protoplasts. Kaplan–Meier curves were performed to compare survival between preincubated or con-injected protoplast with the control group. Pre-incubated protoplasts of *S. parasitica* are not lethal for larvae (*p < 0.1), while the mortality of co-injected larvae is delayed for all tested concentrations but not statistically significant (*p* > 0.1). **b** Progress of an infection with *S. parasitica* exemplarily shown for injections with molecular tweezers only (0 protoplasts, coinjection) and the delay of an infection after coinjection with 500 protoplasts compared to the injection of 500 protoplasts only

As a proof of concept, we have tested if molecular tweezers were able to prevent an infection of *Galleria mellonella* larvae by *S. parasitica*^24^. For this, protoplasts were either pre-incubated with molecular tweezers for 1 h or co-injected with molecular tweezers into larvae (Fig. 6a, b). While larvae injected with protoplasts only died 3 dpi, pre-incubated protoplasts did not cause any mortality and although statistically not significant, protoplast that were co-injected with molecular tweezer showed a delay in mortality of larvae.

### Vesicle release of SpHtp3 is mediated by another effector

**Fig. 7:**
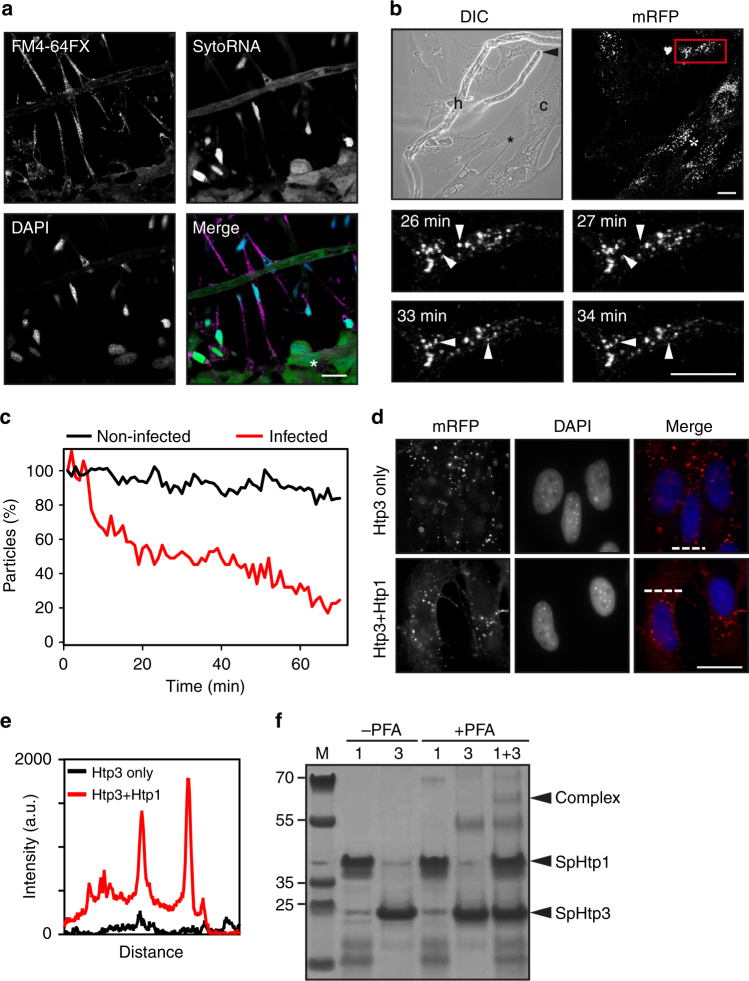
SpHtp3 is released from vesicles with the help of SpHtp1 from *S. parasitica*. **a** RTG-2 cells in direct contact with *S. parasitica* are shrunk with a condensed nucleus. In these cells, no cytosolic RNA (SytoRNA) can be detected and infected cells contain a high amount of vesicles (membrane stain FM4-64FX, see also Fig. 1b). In contrast, cells in close proximity but no direct contact do not show any morphological abnormalities (*). Scale bar: 20 µm (*n* = 3). **b** RTG-2 cells (c) were challenged with *S. parasitica* (h) after 1 h incubation with SpHtp3-mRFP. A hyphal tip (arrowhead, DIC) is attacking an RTG-2 cell. Magnification of the infected cell (red square) at different time points (bottom) show vesicles disappearing within a minute (arrowheads). See also Supplementary Movie 1. In contrast, cells in close proximity but no direct contact to *S. parasitica* contain less disappearing vesicles (*). Scale bar: 20 µm (*n* = 3). **c** Quantification of SpHtp3-mRFP containing vesicles of RTG-2 cells from **b** over time. **d** Vesicle release of SpHtp3-mRFP into the cytosol of RTG-2 cells after pre-incubation with SpHtp1_21–198_-His_6_ at pH 7.5. SpHtp3 accumulates in vesicles of RTG-2 cells after self-translocation (see also Fig. 2d). However, after co-incubation of SpHtp1 with SpHtp3, the number of vesicles in the periphery of the cells is reduced and the cytosolic fluorescence of RFP increased. Pictures were taken with a Zeiss Imager M2. Scale bar: 20 µm (*n* = 2). **e** Fluorescence intensity of SpHtp3-mRFP across the cell as indicated by dashed lines in **d**. **f** In vitro complex formation of recombinant SpHtp1-His_6_ and SpHtp3-His_6_ after cross-link verified by LC-MS/MS (Supplementary Table 2). An additional band, which only appears in the sample with both proteins is highlighted (Complex)

During our infection studies of RTG-2 cells by *S. parasitica*, we could observe the degradation of cytoplasmic RNA visualised by SytoRNA of cells that are in direct contact with hyphae of *S. parasitica*, while the nuclei of infected cells remain intact (Fig. 7a). Hence, we conclude that SpHtp3 or similar, unidentified nucleases must have been translocated into the host cytosol. However, SpHtp3 is the only protein comprising a secretion signal, a predicted bi-functional nuclease domain, without a nuclear localisation signal (which is in line with the cytoplasmic localisation) and up-regulated during infection. To investigate the effect of SpHtp3 under infectious conditions, we have pre-incubated RTG-2 cells with recombinant SpHtp3-mRFP. After SpHtp3 was taken up into vesicles, pre-treated cells were co-incubated with *S. parasitica*. Indeed, in fish cells that are in direct contact with hyphae of *S. parasitica*, vesicles filled with recombinant SpHtp3-mRFP disappeared (Fig. 7b and Supplementary Movie 1). Remarkably, the number of fluorescent SpHtp3 vesicles was only reduced in cells with direct hyphal contact compared to non-infected cells (75% and 17%, respectively) (Fig. 7c), which indicates a cofactor-mediated release of SpHtp3.

Previously we found that SpHtp1 from *S. parasitica* is expressed during early stages of infection and also able to enter host cells^11^. SpHtp1 is an intrinsically disordered protein with a high degree of flexibility, which is characteristic for the mediation of protein–protein interactions. To investigate a potential interaction between SpHtp1 and SpHtp3, we pre-incubated both proteins before performing the translocation assay into RTG-2 cells (Fig. 7d). At pH 7.5, the translocation of SpHtp3 into vesicles is reduced, which is reflected by the low amount of vesicles with a low fluorescence intensity (Fig. 7e). After pre-incubating with SpHtp1 vesicles, the periphery of the cell disappear and the cytosolic RFP fluorescence is increased. This might indicate a potential role of SpHtp1 in the uptake of SpHtp3 at a neutral pH (pH 7.5) and its release from vesicles. Indeed, co-incubation of recombinant SpHtp1 and SpHtp3 (mRFP- or His-tagged) in vitro, resulted in an additional band for a cross-linked SpHtp1–SpHtp3 complex, which was confirmed by LC-MSMS analysis (Fig. 7f, Supplementary Table 2).

## Discussion

**Fig. 8:**
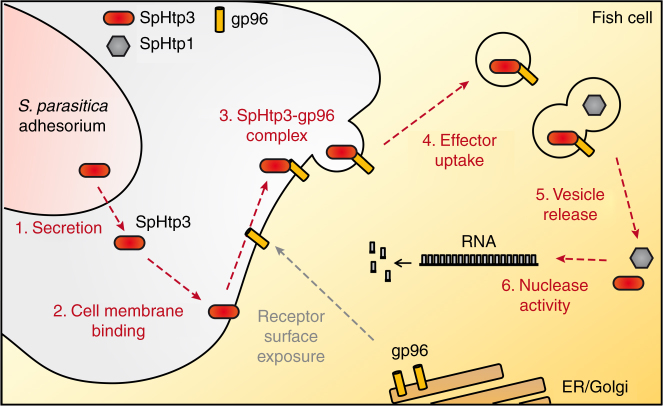
Model for the self-translocation of SpHtp3 into host cells and its vesicle release. The fish-pathogenic oomycete *S. parasitica* secretes several effector proteins during different stages of infection. The nuclease SpHtp3 is secreted in the later stages of infection. *S. parasitica* acidifies the pH of its environment, which likely leads to the exposure of a gp96-like protein to the host cell surface. The gp96-like protein is working as a receptor and mediates the translocation of SpHtp3 via lipid rafts into the cell. Finally, SpHtp3 is released from vesicles with the help of other effector proteins, as SpHtp1, into the cytosol where it is functionally active as a nuclease

For the successful establishment of an infection, plant-pathogenic oomycetes use a broad spectrum of secreted effector proteins to promote the infection process by mediating invasion and suppressing host immune responses^3,5^. Currently, the pathogen-independent self-translocation of effector proteins secreted by plant-pathogenic oomycetes is unclear^9,25^. The present study with SpHtp3 from the fish-pathogenic oomycete *S. parasitica* reveals a potential model for effector translocation from oomycetes into host cells (Fig. 8). Interestingly, uptake studies with the SpHtp3 homologue from the plant-pathogenic oomycete *P. sojae* (PsHtp3) into fish cells confirmed the pathogen-independent self-translocation also in non-host cells (Supplementary Fig. 4a). Homologues of the gp96 receptor are also present in several plant species with an identity of 51%^26^, but a potential role for the uptake of effector proteins into plant cells remains to be investigated.

The largest group of effector proteins of the well-known plant-pathogenic oomycetes, *P. sojae* and *P. infestans*, is characterised by a highly conserved RxLR motif (Arg–Xaa–Leu–Arg), which is thought to play a role during the secretion and/or translocation of effectors into plant cells^7,13,27–31^. However, the RxLR motif of SpHtp3 from *S. parasitica* is not involved in the translocation process. Instead, the C-terminus presumably interacts with the negatively charged cell membrane of the host, like the positively charged lysine patches on the effector domains of AVR3a from *P. infestans* and AVR1b from *P. sojae*^10,12,32^. Regarding the uptake process, the attachment and invasion of *Escherichia coli* to human endothelial cells (HBMEC) are two independent and different processes mediated by the same motif^33^ similar to our observation with SpHtp3.

After binding to the cell, SpHtp3 is taken up into fish cells mediated by a gp96-like protein located in lipid rafts, which is a very ancient evolutionary way of translocating molecules^34^. The gp96-mediated translocation has a low affinity but a high capacity, which is perfectly suited for the uptake of a huge reservoir of effectors secreted by pathogens^35^. This is supported by a large number of vesicles, which can be observed in single cells infected with *S. parasitica* (Figs. 1b and 6b). Normally, gp96 is a chaperone of the Hsp90 family located in the ER but is also found exposed to the cell surface^36–42^. The environmental acidification by *S. parasitica* (Supplementary Fig. 4b) and the reduction of the extracellular space during an infection result in an increased exposure of gp96 to the cell surface (Fig. 4h)^42–44^. The ubiquitous expression of gp96 in several tissues as well as across a broad range of species results in a potential receptor function for several pathogens ranging from bacteria like *E. coli*^46,47^, *Listeria monocytogenes*^48,49^ or *Clostridium difficile*^50,51^ to fungi like *Candida albicans*^52^ and the *vesicular stomatitis virus*^45^.

gp96 occurs naturally as a mixture of non-glycosylated and hyper-glycosylated molecules with up to five glycosylation sites inserted probably by a sequential mechanism^53–55^. It is postulated that the deglycosylated form has a higher affinity to substrates^55^. Thus, we speculate that the lower spot of gp96 after SpHtp3 incubation on 2D-PAGE (Fig. 4c) is the deglycosylated moiety of gp96, which has bound SpHtp3 and was relocalised into the cell. Another posttranslational modification of gp96 is the phosphorylation of tyrosine residues by the Fyn kinase in the ER, which is important for the shuttling of gp96 between the ER and the cell membrane^53,56,57^. Accordingly, the inhibition of tyrosine kinases by genistein leads to the intracellular trapping of gp96 and in line with our studies, the SpHtp3 translocation is clearly reduced (Supplementary Fig. 4c). During the re-uptake of gp96 with or without a ligand, gp96 interacts with sulfated sites of lectins (OS-9) and heparin/HSPG^35,58^. Hence, after treating cells with the sulfation inhibitor NaClO, the binding of gp96 to the cell surface is abolished^35^ in the same way as the uptake of SpHtp3 into fish cells (Supplementary Fig. 4d). Interestingly, SpHtp1 also translocates into host cells in an O-sulfation dependent manner^11^. However, both effector proteins are expressed during different stages of infection and are clearly using different pathways into the host cell (Fig. 4a vs. Supplementary Fig. 3b). Therefore, we speculate that both effectors attach to the host membrane by ionic interactions, but are using different receptor molecules to enter the host cell.

Our research has given novel insights into how a fish-pathogenic oomycete establishes an infection. The single most limiting factor for sustainable expansion of fish farming is disease. Indeed, *S. parasitica* is responsible for major losses in the aquaculture industry^1,2^. Therefore, a detailed understanding of the infection processes at the molecular level is very important for the development of new control strategies against oomycetes that will address global challenges in sustainable food security^4^.

## Methods

### Cloning of SpHtp3 constructs

*SpHtp3*_*21–211*_ lacking the putative N-terminal signal peptide was amplified from a cDNA of *S. parasitica* using the KOD Hot start polymerase (Novagen) according to the manufacturer’s protocol. Primer: fw (+ *Nde*I) 5′-TACAGTCATATGCTGCTCATCCCGCACCTCC-3′ and rv (+ *Eco*RI) 5′-ATCGATGAATTCATTTGTACTGCGCAGGCGTCTCG-3′ [58 °C]. PCR fragment was cloned into the pGEM-T easy vector (Promega) according to manufacturer’s protocol. The pGEM-T easy vector and pET21b or pET21b-mRFP (Novagen^11^) were digested with *Nde*I and *Eco*RI for subsequent sub-cloning. The N-terminal moiety of SpHtp3 containing the RxLR sequence (21–55 aa) was obtained by amplifying the *SpHtp3*^21–55^ fragment from pET21b containing SpHtp3^21–211^. Primer: fw (+ *Nde*I) 5′-TACAGTCATATGCTGCTCATCCCGCACCTCC-3′ and rv (+ *EcoR*I) 5′-GAGCTCGAATTCATGACGACCTTGCCGCGGAGT-3′ [58 °C]. The *SpHtp3*_21–55_ fragment was then cloned into pET21b-mRFP using the *Nde*I/*Eco*RI cut sites. The RTLR/GTLG mutant was purchased from GenScript and sub-cloned into pET21b-mRFP. The K208A/R210A double mutant of SpHtp3 was prepared with the Q5® Site-Directed Mutagenesis Kit (#E0554, NEB). All constructs were verified by sequencing (Source Bioscience).

### Expression and purification of recombinant proteins

Purification procedure is exemplarily shown for SpHtp3-mRFP. Various SpHtp3 constructs were transformed into *E. coli* Rosetta gami B (DE3, pLys; #71137, Novagen) cells. Cells were inoculated and grown in LB-media to an OD600 of 0.8 and protein expression was subsequently induced with 1 mM IPTG for 6 h at 37 °C. Cultures were centrifuged (10 min, 15,000×*g*, 4 °C) and pellets re-suspended in 40 ml 25 mM NaP_i_ buffer (pH 7.5) supplemented with 250 U benzonase (#E1014, Sigma), two tablets of protease inhibitor (#04693159001, Roche) and 1 mM PMSF. Cell lysis was done with 0.1 g lysozyme (#62971, Fluka) for 20 min at 4 °C and successive French-Press. Cell debris and aggregates were pelleted by centrifugation (1 h, 48,000×*g*, 4 °C) and the supernatant applied to a QAE agarose column equilibrated with 25 mM NaP_i_ (pH 7.5). The flow-through was subsequently loaded onto a Ni-NTA agarose column, followed by one wash step with 25 mM NaP_i_ (pH 7.5) and another one with 25 mM NaP_i_ (pH 7.5) supplemented with 30 mM imidazole. SpHtp3-mRFP was eluted with 300 mM imidazole. The elution fraction was then applied to a SO_3_^−^ column. Two washing steps followed: 25 mM NaP_i_ (pH 7.5) and 25 mM NaP_i_ (pH 7.5) with 50 mM NaCl. SpHtp3 was eluted by 25 mM NaP_i_ (pH 7.5) containing 500 mM sodium chloride and 10 mM magnesium sulphate for protein stabilisation. Fractions containing SpHtp3-mRFP were pooled and stored at −20 °C.

### Electron microscopy (SEM and TEM)

For electron microscopy, infected RTG-2 fish cells were prepared as described in ‘In vitro infection assays with *S. parasitica*’. Samples were fixed in 3% glutaraldehyde in 100 mM NaP_i_ (pH 7.4) for 24 h. Afterwards, samples were washed with 100 mM NaP_i_ (pH 7.4), three times and 5 min each. Subsequently, samples were processed in an automated routine tissue processor Leica EM TP (Leica Microsystems, Vienna, Austria) comprising following steps. Post-fixation in 1% (v/v) osmium tetroxide (OsO_4_; aqueous solution) for 1 h preceded by three washing steps with 100 mM NaP_i_ (pH 7.4), 5 min each with distilled water and an extra wash step for 30 min. Next, samples were dehydrated in increasing concentrations of ethanol (30, 70, 90 and 100% (v/v); 30 min each).

For TEM, the dehydration is followed by three incubations in acetone for 1 h. Samples were then incubated in increasing concentrations of epoxy/acetone (1/1, 6/1 and 100% epoxy) for 1 h, 6 h and 24 h, respectively, before embedding the samples in labelled capsules with freshly prepared resin, leaving the resin to polymerise for 48 h at 60 °C. Ultra-thin sections (70–80 nm) were cut with a Leica EM UC6 ultramicrotome (Leica Microsystems, Vienna, Austria) and mounted on 200-mesh uncoated copper grids. Grids were stained with 2% (v/v) uranyl acetate and 0.5% (v/v) lead citrate on an automated contrasting instrument Leica EM AC20 (Leica Microsystems, Vienna, Austria). Finally, the grids were analysed at 80 kV using a JEM-1400 Plus (JEOL Ltd., Tokyo, Japan) transmission electron microscope equipped with an AMT UltraVUE camera.

For SEM, the dehydration with the ethanol series is followed by a speed critical point drying with hexamethyldisilazane (HMDS) for 20 min and subsequently air drying at RT overnight. Dried samples were coated in gold using an EMitech K550 sputter coater. Coated samples were viewed in a Zeiss EVO MA10 SEM at 10 kV.

### Live cell imaging

Cell culture maintenance for RTG-2 (ATCC CCL-55), RTL-W1^41^, RTGill-W1 (ATTC CRL2523) was done according to the manufacturer’s or publisher’s instructions. L-15 medium (Gibco) was supplemented with 10% FBS (#PS/437003224, Sigma), 100 units ml^−1^ Penicillin and 100 μg ml^−1^ Streptomycin. Wells containing coverslips with attached cells were washed 3× with medium. For the NaClO_3_ treatment (sulfation inhibition), cells were incubated with 70 mM NaClO_3_ for 48 h before incubation with SpHtp3 constructs. Cells were then incubated with 3 μM of various mRFP-tagged proteins (SpHtp3_21–211_-mRFP (fl), SpHtp3_21–55_-mRFP, SpHtp3^RTLR/GTLG^-mRFP) for 1 h at 18 °C. If not stated otherwise, incubation was done with acidified media (pH 5.5). For stabilisation of proteins, the medium was supplemented with 60 mM MgSO_4_. After incubation, cells were washed 3× with Hanks’ Balanced Salt Solution (HBSS) and coverslips were transferred to optical petri-dishes (Ø 3 cm) and covered with cell culture medium. Analysis was done on a Zeiss LSM 510 confocal microscope equipped with a water dipping lens. All images were recorded with the same settings (optical slice = 2 μm; ex: 543 nm; detector gain: 750; filter: LP 560 nm).

### Fixed cell imaging

Cell culture maintenance fish cell lines see ‘Live cell imaging’. For A549 (ATCC CCL-185), cells were maintained according to the manufacturer’s instruction in DMEM (Gibco) supplemented with 10% FBS, 100 units ml^−1^ Penicillin and 100 μg ml^−1^ Streptomycin. Cells were plated onto glass coverslips and incubated to a confluence of 80%. Cells were pre-incubated with various inhibitors (dynasore (#14062, Cayman Chemical Company), Brefeldin A (# 00-4506-51, e-bioscience) or 10 mM nystatin (#475921, Calbiochem)) for 1 h at 18 °C. Afterwards, 3 μM of various protein constructs (SpHtp3-mRPF wt, SpHtp3-mRFP^K208A/R210A^ or PsHtp3-mRFP wt) or a C-terminal peptide of SpHtp3 (FITC)Ahx-KRETPAQYKARKMNSSSVD (Ahx = aminohexanoic acid linker, CASLO, Denmark) were incubated for 1 h at 18 °C. If not stated otherwise, incubation was done with acidified media (pH 5.5). After incubation, cells were washed 3× with HBSS and fixed with 4% ice-cold PFA in PBST (PBS + 0.1% tween) for 15 min at RT. Residual PFA was removed with 5× washing steps with PBS. Cells were mounted with Vectashield with DAPI (# H-1200, Vector Laboratories). Samples were analysed by a Zeiss LSM710 confocal microscope or an epifluorescence microscope (Zeiss Imager M2 with metal halide light source). For quantification, mean intensity or vesicles per cell were counted with ImageJ. A maximum of five cells were analysed from the same picture. Bars denote s.e.m. of 50 cells counted per sample.

For immunolocalisation of gp96 in human A549 cells, cells were plated on glass coverslips and incubated with 2.5 μg α-gp96 antibody (#H-212, Santa Cruz Biotechnology) for 30 min at 18 °C before adding 3 μM SpHtp3-mRFP for another 1 h at 18 °C. Cells were subsequently washed 3× with HBSS and fixed with 4% ice-cold PFA in PBST (PBS + 0.1% tween) for 15 min at RT. Residual PFA was removed with 5× washing steps with PBS. Hydrophobic areas were blocked with 10% goat serum (#S-1000, Vector Laboratories) in PBS for 1 h at RT followed by three washing steps with PBS. 4 µg of a secondary anti-rabbit antibody (1:1000, #A31629, Invitrogen) was diluted in PBS (3% goat serum) and incubated for 1 h at RT. Remaining antibody was removed by a washing step (1× PBS, 3× PBST) and slides covered with Vectashield mounting medium with DAPI.

### Flow cytometry (FACS)

Equalised cell suspensions were grown to confluent layers (2 × 10^6^ cells, 25 cm^2^ flask). Cells were washed 3× with PBS and subsequently incubated with 3 μM of various mRFP-tagged proteins (SpHtp3_21–211_-mRFP (fl), SpHtp3_21–55_-mRFP, SpHtp3^RTLR/GTLG^-mRFP) for 1 h at 18 °C at pH 5.5 if not otherwise stated. After incubation, cells were washed 3× with PBS and incubated for 5 min with the DNA stain TO-PRO-3 iodide (#T3605, Invitrogen, 1:1000, ex: 633 nm, em: 660 nm) dissolved in L-15 medium. Residual DNA stain was removed with another 3× washing steps with PBS. Cells were detached with 1 ml trypsin (0.5 mg ml^−1^) and filtrated through a cell strainer. mRFP fluorescence (ex: 488 nm, em: 610 nm) was quantified on an LSR II flow cytometer (BD Bioscience). For final analysis a homogenous population of cells that were TO-PRO-3 iodide negative was selected (~ 80% of 10,000 counted events). Data analysis was carried out using FlowJo v 7.6.

### siRNA-mediated knock down of gp96

Protein level of gp96 in human A549 cells was reduced by a commercially available and verified siRNA (#4392420, s14375, Ambion, sense 5′-GGUCAGAGCUGACGAUGAAtt-3′, antisense 5′-UUCAUCGUCAGCUCUGACCga-3′). The knock down was performed according to the manufacturer’s instruction for a 25 cm^2^ flask. For this, solution A (750 μl Opti-MEM + 7.89 μl RNAiMAX (#13778150, Life Technologies)) and solution B (750 μl Opti-MEM + 7.89 μl siRNA (s14375)) were briefly vortexed. Subsequently, solutions A and B were mixed, vortexed and incubated for 30 min at RT. As a negative control, solution B was prepared without siRNA. A549 cells were incubated for 48 h with the anti-gp96 siRNA without a medium change. After 2 days, A549 cells were washed twice with HBSS and detached with trypsin (0.5 mg ml^−1^). 3.6 × 10^4^ cells ml^−1^ were plated on glass coverslips and used to investigate the translocation of SpHtp3-mRFP. The rest of the cells were used to confirm the knock down on protein level by western blot (originals are included in the Supplementary Information).

### Nuclease activity assay

For determination of DNA and RNA degrading activity, 3.4 μM SpHtp3-mRFP or SpHtp3-His_6_ were incubated with 150 ng of linearised pET21b plasmid DNA or 2 µg of RTG-2 cell total RNA (isolation with TRI Reagent (#T9424, Sigma-Aldrich) according to the manufacturer’s protocol) in a total volume of 25.5 μl for 5 min at RT. Control samples contained buffer only. For the inhibition of potential RNAase contamination from the purification process, 7.5 µM RiboLock (#EO0381, Thermo Fisher) were added to the reaction where indicated (Thermo Scientific). Proteins were removed by heating the samples at 65 °C for 5 min. Samples were analysed on a 1% agarose gel (originals are included in the Supplementary Information).

### Kinetic measurements of ribonuclease activity

Real-time fluorescent monitoring of ribonuclease activity was performed at RT using the RNaseAlert^(R)^ Lab Test Kit (#AM1964, Applied Biosystems). 80 nM of SpHtp3-mRFP or SpHtp1-mRFP (as a negative control) were diluted in a total volume of 600 μl of 25 mM Hepes pH 7.5. Protein constructs were dialysed prior to measurements for 24 h against 25 mM Hepes. Where indicated, 25 mM MgSO_4_, MgCl_2_ or NaSO_4_ were added to the reaction. The reaction was started with 25 pmol of a fluorescent substrate (excitation: 490 nm; emission: 520 nm) and subsequently monitored by a FLS920 fluorescence spectrometer (Edinburgh Instruments) using a 4 × 4 mm^2^ stirred cuvette.

### In vitro infection assays with *S. parasitica*

Culture conditions and zoospore/cyst production of *S. parasitica* (CBS223.65, originally isolated from young pike (*Esox lucius*)) was described previously^1^. RTG-2 cells were grown to 70% confluence on glass coverslips. For the infection of RTG-2 cells with *S. parasitica*, 3750 zoospores/cysts were diluted in HBBS supplemented with 10% FBS and 30% L-15 medium. Zoospores/cysts were added to the cells and incubated for 14 h at 24 °C.

For bright field microscopy, infected cells were visualised with an Evos XL light microscope. For fluorescence microscopy, cells were washed after co-incubation of cells and spores 3× with HBSS and fixed with 4% ice-cold PFA in PBST (PBS + 0.1% tween) for 15 min at RT. Residual PFA was removed with 3× washing steps with PBS and RTG-2 cell/*S. parasitica* hyphae were stained with SytoRNA (1:10,000, #S32703, Life Technologies) for 20 min at RT in the dark. Remaining dye was removed with 3× washing steps with PBS. Successively, membrane was stained by FM4-64FX (2 nM, #F34653, Life Technologies) for 5 min on ice in the dark. Remaining dye was removed with 3× washing steps with PBS. Cells were mounted with Vectashield with DAPI. Samples were analysed by a Zeiss LSM710 confocal microscope.

Vesicular release of SpHtp3: RTG-2 cells were grown to confluence and incubated with 3 µM SpHtp3_21-211_-mRFP for 1 h at 18 °C. Cells were washed 3× with L-15 medium and once with HBSS (Gibco) to remove non-translocated protein as well as remaining nutrients. Subsequently, 1 ml of zoospores/cysts (~3750 cells ml^−1^) of *S. parasitica* in HBSS supplemented with 3% FBS and 30% L-15 medium were added to the cells. Cells were co-incubated with the zoospores/cysts for another 3 h at 18 °C and SpHtp3-mRFP was monitored by confocal microscopy with a Zeiss LSM 510 confocal microscope equipped with a water dipping lens. Translocation and release from vesicles of SpHtp3_21–211_-mRFP was investigated for 70 min at RT. In total 70 frames were taken, each with a stack of 10 optical slices (z-series) to detect also moving vesicles. Shown are the Z-projections for the time step as indicated which were also used to analyse the decreasing mRFP fluorescence over time with ImageJ. Number of particles was counted for the infected cell compared to all non-infected cells.

### In vivo infection assay with *Galleria mellonella*

An infection assay with *G. mellonella* was used to determine the effect of the molecular tweezers on the virulence of *S. parasitica*^24^. Five larvae per group were injected with 1000, 500, 250 or 0 protoplast as indicated. Control larvae were injected with protoplasts only. One group of protoplast was preincubated with 5 mM tweezers at RT for 1 h, before injection. In another group, 5 mM molecular tweezers were injected into larvae 1 h before protoplast were injected. Larvae were incubated at 24 °C and the progress of infection monitored every 12 h.

### Protein cross-linking

In order to detect a direct interaction, 10 μg of SpHtp1 and 10 μg of SpHtp3 or each protein alone were pre-incubated in PBS (total: 15 μl) for 15 min at RT. For cross-linking, 15 μl ice-cold 4% PFA/PBS (final concentration: 2%) was added and incubated for 10 min at RT. The reaction was stopped with 7 μl Laemmli loading dye and subsequent heating to 65 °C for 10 min. Complex formation was investigated by SDS-PAGE (originals are included in the supplementary information) and confirmed by LC-MS/MS.

### CD spectroscopy

Far-UV CD spectra were recorded with a Jasco J-710 spectropolarimeter (Jasco, Gross-Umstadt) with 0.2 mg ml^−1^ SpHtp3-His_6_ in 50 mM NaP_i_ buffer (pH 5.0, 6.0 and 7.0) at 21 °C in 1 mm cuvettes. A buffer baseline was subtracted and units are given as ellipticity. Secondary structure content was evaluated using the CDSSTR algorithm.

### pH measurements during *S. parasitica* growth

In order to observe the effect of the growth of *S. parasitica* on the environmental pH, liquid cultures were inoculated with agar plaques and their pH measured after various time points. 30 ml of PG-1 medium (3 g l^−1^ peptone, 6 g l^−1^ glucose) was inoculated with two agar plugs each (covered with mycelium of *S. parasitica*). Tubes were incubated at RT and the pH measured on a daily basis for 7 days. To prevent contamination, for each time point a separate tube was prepared and discarded after measurement of the pH. For each time point, three replicates were measured. As a control, PG-1 medium was inoculated with sterile agar plugs. Per time point average and standard deviation were calculated.

### NMR spectroscopy and structure calculation

NMR experiments were performed on a 700 MHz Ultrashield NMR spectrometer (Bruker, Ettlingen, Germany) equipped with a cryoprobe (Bruker Biospin). 2 mM of the C-terminal peptide of SpHtp3 (KRETPAQYKARKMNSSSVD, FITC-coupled) or the corresponding mutant was dissolved in 600 μl (90% H_2_O, 10% D_2_O) 50 mM NaP_i_ buffer (pH 7.3). Standard 2D spectra (NOESY, TOCSY, COSY) were used for backbone assignment. For titration experiments ^1^H-1D spectra were recorded with 128 scans. Data were processed with Topspin 3.0 (Bruker).

Titration experiments were performed at 25 °C by recording spectra after each stepwise addition of different concentrations of molecular tweezers as indicated to 2 mM pf SpHtp3 peptide. Intensity changes or shifts of signals indicate a binding between both interaction partners.

NMR-based structure calculation was performed using the software CYANA 2.1. Manually picked NOESY cross-peaks from a ^1^H–^1^H NOESY spectrum of SpHtp3 peptide were automatically assigned by CYANA and used for calibration and successive calculation of distance constraints. Dihedral angles were predicted from assigned H atoms by TALOS+. Distance and angle constraints were used for seven cycles of combined automated NOESY assignment and structure calculation, followed by a final structure calculation. For each structure, 200 conformers were calculated using the standard simulated annealing schedule with 10,000 torsion angle dynamics steps per conformer.

### Homology modelling

Homology models were generated using the YASARA Structure suite with parameters given in Table 1. Molecular dynamics simulations were performed applying a YAMBER2 force field with the YASARA Structure suite using default parameters and 1 fs time steps. Electrostatic potentials were calculated using the Adaptive Poisson-Boltzmann Solver (APBS) applying the YAMBER2 force field.

**Table 1:** Parameters for homology modelling with YASARA

Parameter	Value
Modelling speed (slow = best)	Slow
Number of PSI-BLAST iterations in template search (PsiBLASTs)	1
Maximum allowed (PSI-)BLAST *E*-value to consider template (EValue Max)	0.5
Maximum number of templates to be used (Templates Total)	5
Maximum number of templates with same sequence (Templates SameSeq)	1
Maximum oligomerization state (OligoState)	1 (monomeric)
Maximum number of alignment variations per template (Alignments)	5
Maximum number of conformations tried per loop (LoopSamples)	50
Maximum number of residues added to the termini (TermExtension)	30

### Statistics

Live cell imaging was repeated at least three times for each condition and quantitative analysis was performed by FACS analysis. Experiments with fixed cells were performed at least three times (Fig. 4f twice) with the same result. Quantitative analysis is shown for one experiment of 50 cells from at least 10 different visual fields exemplarily. Comparison of two groups was done with *t*-test and for more than two groups, one way-ANOVA was applied. The real-time nuclease activity was measured twice (Fig. 2c) and the live cell imaging infection assay was performed twice (Fig. 7b). Error bars indicate s.e.m. A log rank test was performed for the survival curves of the in vivo infection experiment (co-injection/preincubation vs. control). The results show an increase in survival rate of co-injected tweezers, but this was not statistically significant (*p* > 0.1).

### Data availability

The authors declare that data supporting the findings of this study are available within the paper and its supplementary information files. Assignment and calculation data that support the findings of the structure of the C-terminal peptide of SpHtp3 are available from the corresponding author upon request.

## Electronic supplementary material

Supplementary Information

Description of Additional Supplementary Files

Supplementary Movie 1
