# Supplementary material for: Cell entry of a host-targeting protein of oomycetes requires gp96
Source: Nat Commun. 2018 Jun 14;9:2347. doi: 10.1038/s41467-018-04796-3 (PMC6002402; doi:10.1038/s41467-018-04796-3)
Supplement: Supplementary file 1 — Supplementary Information [file 41467_2018_4796_MOESM1_ESM.pdf]

SUPPORTING INFORMATION

Cell entry of a host targeting protein of oomycetes requires gp96

Trusch et al.

Trusch F, Loebach L, Wawra S, Durward E, Wuensch A, Iberahim NA, de Bruijn I, MacKenzie K, Willems A, Toloczko A, Diéguez-Uribeondo J, Rasmussen T, Schrader T, Bayer P, Secombes CJ, van West P

Content:

Supplementary Figure 1 page 2

Supplementary Figure 2 page 3

Supplementary Figure 3 page 4

Supplementary Figure 4 page 5

Supplementary Table 1 page 6

Supplementary Table 2 page 7

Original gels and blots page 8

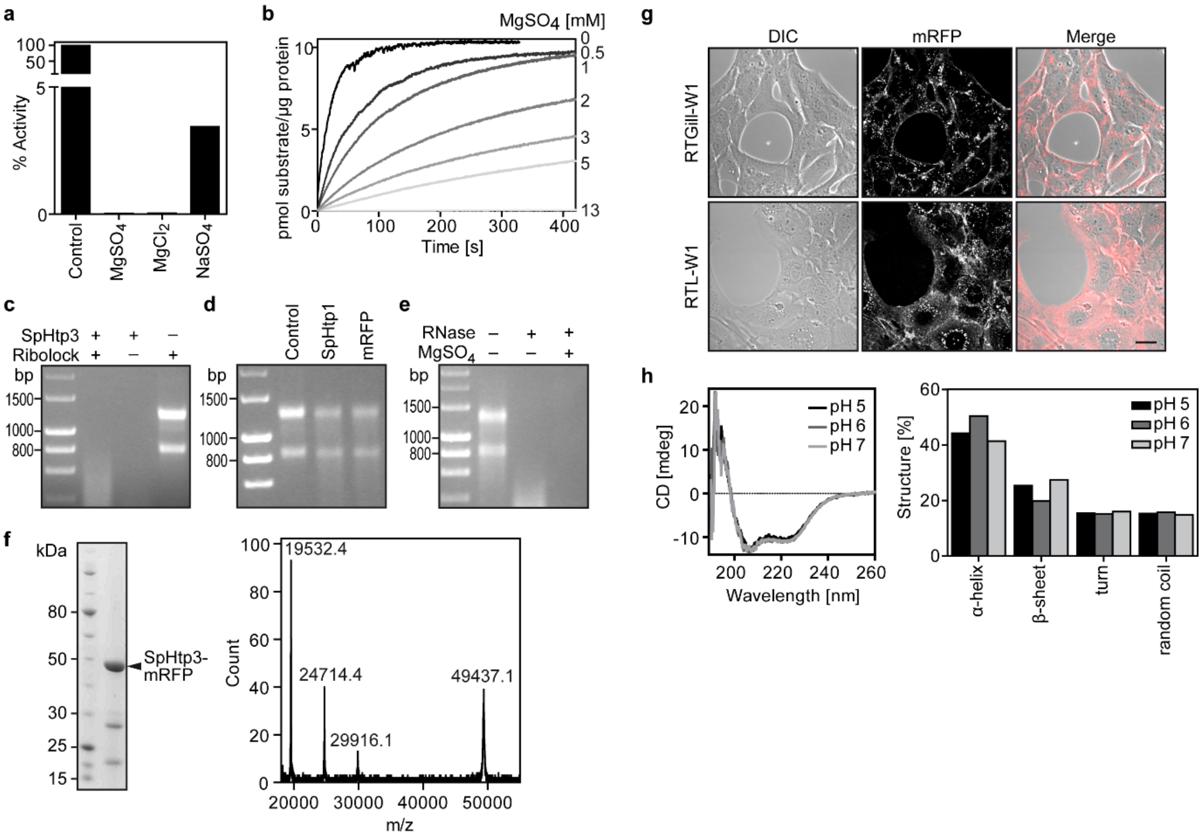

Supplementary Figure 1. Nuclease activity and self-translocation of SpHtp3. (a) Impact of different salt ions ($MgSO_4$, $MgCl_2$, $NaSO_4$, 25 mM each) on the ribonuclease activity of SpHtp3. Enzyme activities of different salt treatments were normalized to a control sample without additional salts (25 mM Hepes; 100 % activity). **(b)** Real time fluorescent monitoring of the ribonuclease activity of SpHtp3 at different $MgSO_4$ concentrations as indicated using a fluorescently tagged RNA substrate under the control of a quencher (RNaseAlert(R), Applied Biosystems). **(c)** RNase activity visualized by an agarose gel. RNA degradation cannot be inhibited by Ribolock, which excludes potential impurities from the purification process of SpHtp3. (n=2) **(d)** Proteins (SpHtp1-mRFP and mRFP) which were purified in the same way as SpHtp3 do not show any nuclease activity. (n=2) **(e)** RNA degrading properties of RNase A. In contrast to SpHtp3 the activity of RNase A cannot be inhibited by 40 mM $MgSO_4$. (n=2) **(f)** Purity of recombinant proteins, overexpressed in and purified from E. coli, used in this study exemplarily shown for SpHtp3-mRFP confirmed by SDS-PAGE (left) and MALDI-TOF (right). Analysis reveals pure SpHtp3-mRFP (theoretical mass: 49.4 kDa) with two degradation bands from mRFP (confirmed by LC-MS/MS). **(g)** Self-translocation of SpHtp3-mRFP into living epithelial RTGill-W1 cells (top) and RTL-W1 cells (bottom) after 1 h incubation at 18 °C. (n=3) **(h)** CD spectra of C-terminal His_6-tagged SpHtp3 in NaP_i buffer at pH 5.0, 6.0 and 7.0 (left). Corresponding prediction of secondary structure elements from CD spectra using the CDSSTR tool (right).

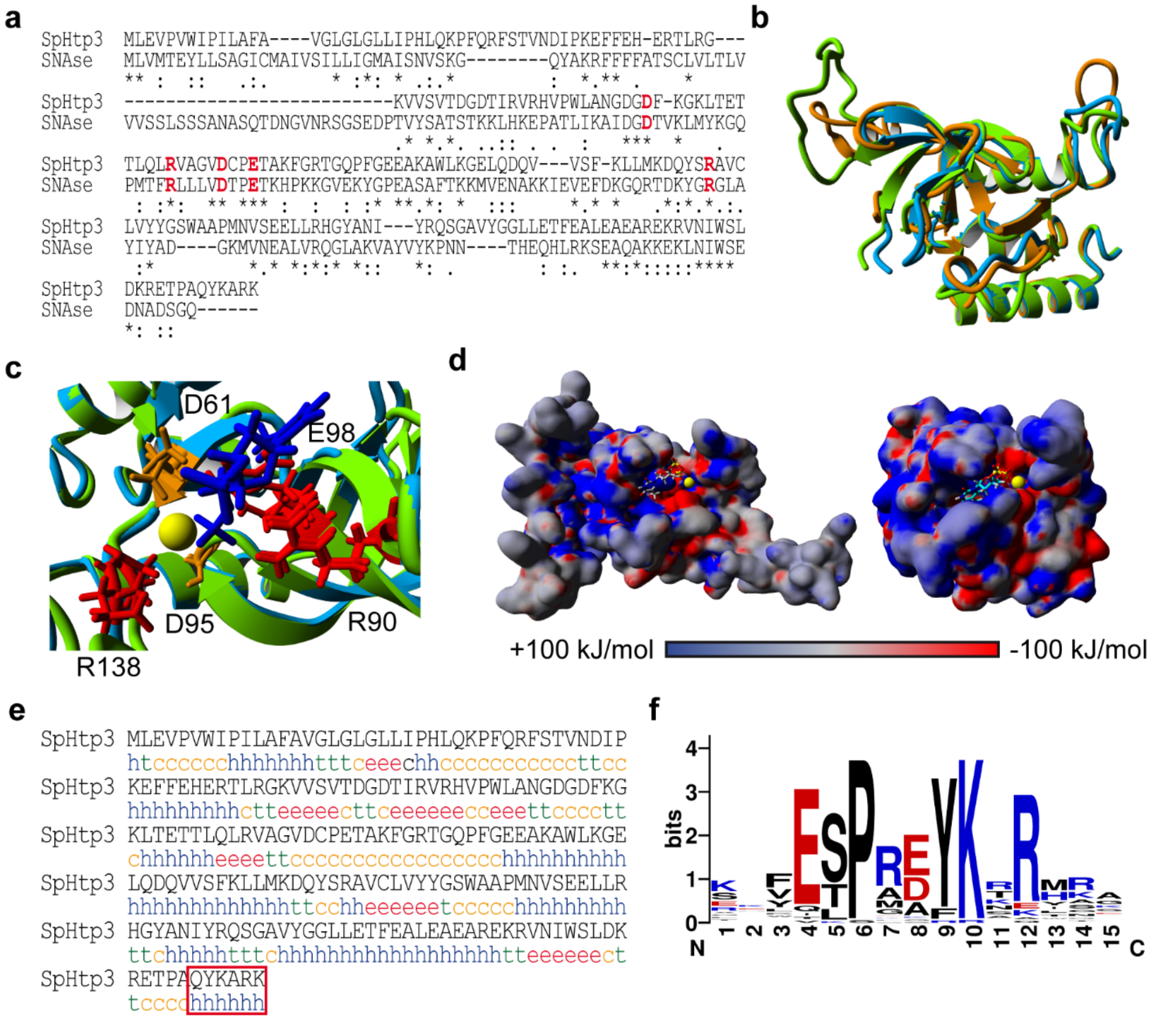

Supplementary Figure 2. SpHtp3 comprises dual nuclease activity. (a) Sequence alignment of SpHtp3 from S. parasitica and SNase from S. aureus (30% identity) with Clustal Omega. Active side residues are highlighted. **(b)** Overlay of the X-ray structure of SNase (blue, PDB-ID: 1NUC), the YASARA-based SpHtp3 model (green) and Phyre2-based SpHtp3-model (orange) shows a reasonable similarity between both nucleases despite the low sequence identity. **(c)** Magnification of the active site of an overlay of the structures of SNase (blue) and the YASARA-based SpHtp3 model (green). Residues of the Ca^{2+} binding site (D61 and D95, orange), the catalytic triade (R90, E98 and R138, red) and ligands (Ca^{2+}: yellow, nucleotide analogue: blue) are labelled according to the SpHtp3 model. **(d)** Electrostatic surface potential presentation of SpHtp3 (left) compared to SNase (right) showing the active site. Positive, neutral, and negative charges are displayed in blue, gray, and red as indicated, respectively. **(e)** Secondary structure prediction of $SpHtp3_{21-211}$ by SOPMA. The C-terminal helix is highlighted. c: random coil, e: extended strand, h: helix, t: turn. **(f)** Weblogo of the conserved C-terminus of SpHtp3 and SpHtp3-like nucleases from 41 different pathogenic fungi and oomycetes (Supplementary Table 1).

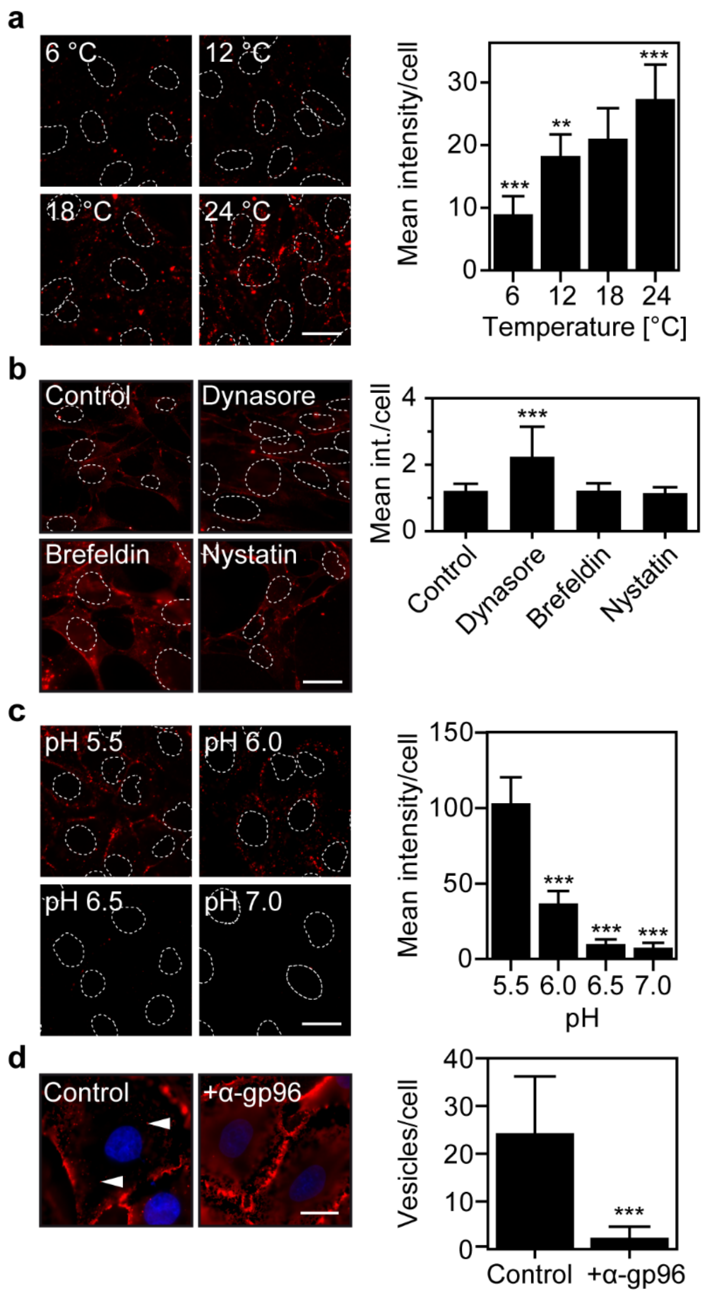

Supplementary Figure 3. Conditions for the successful uptake of SpHtp3. (a) Uptake of SpHtp3-mRFP into RTG-2 cells after 1 h of incubation at different temperatures as indicated (left) and quantitative analysis (right). Nuclei are indicated by dashed lines. Error bars denote s.e.m. (cells: 50). *** $p < 0.001$ (one way ANOVA). Scale bar: 20 µm. (n=3) **(b)** Uptake inhibition of SpHtp1-mRFP into RTG-2 cells pre-incubated for 1 h with the inhibitors dynasore, brefeldin A or nystatin (top) and respective quantification (bottom). Nuclei are indicated by dashed lines. Error bars denote s.e.m. (cells: 50). *** $p < 0.001$, ** $p < 0.01$ (one way ANOVA). Scale bar: 20 µm. (n=3) **(c)** Uptake of SpHtp3-mRFP into human A549 cells after 1 h of incubation at different pH values as indicated (left) and quantitative analysis (right). Nuclei are indicated by dashed lines. Error bars denote s.e.m. (cells: 50). *** $p < 0.001$ (one way ANOVA). (n=3) **(d)** α-gp96 antibody mediated uptake inhibition of SpHtp3-mRFP into human A549 cells after 1 h incubation at pH 5.5. The number of vesicles (arrowheads) is significantly reduced in the antibody-treated sample. Pictures were taken with a Zeiss Imager M2. Error bars denote s.e.m. (cells: 50). *** $p < 0.001$ (t-test). Scale bar: 20 µm. (n=3)

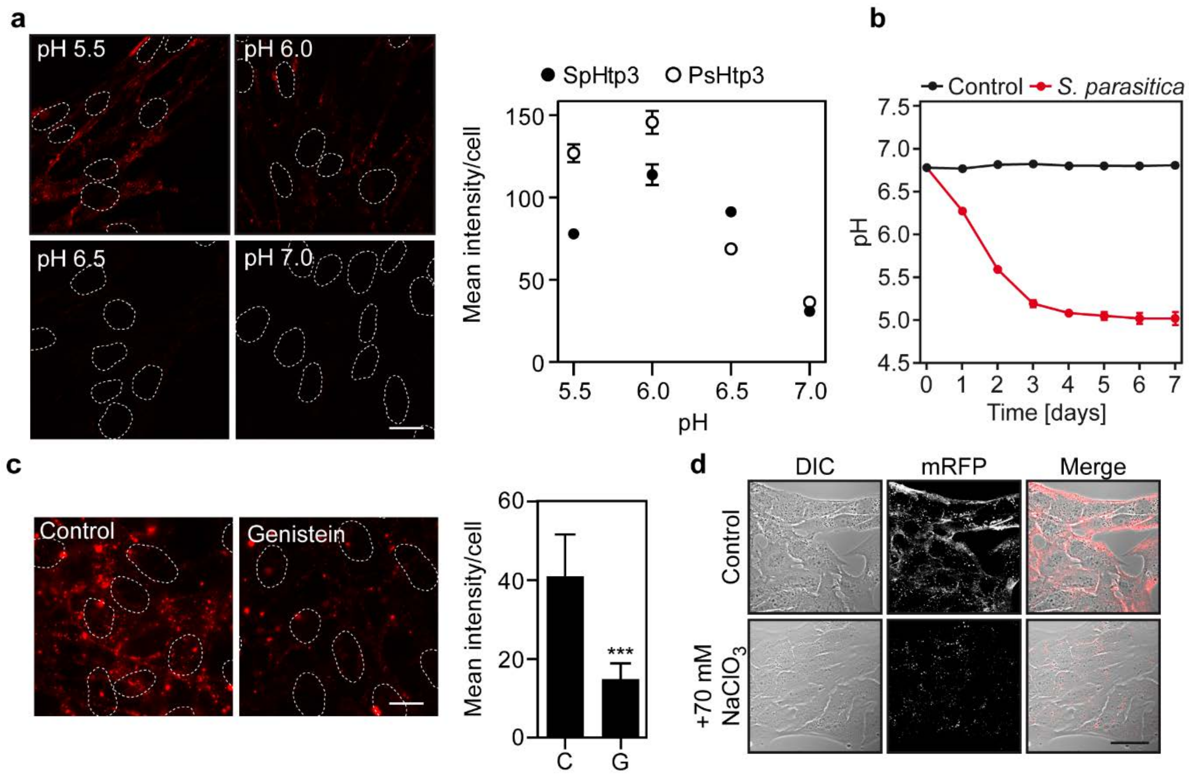

Supplementary Figure 4. The SpHtp3 uptake is dependent on the pH on several levels. (a) Uptake of the SpHtp3 homologue PsHtp3-mRFP from P. sojae into RTG-2 fish cells after 1 h of incubation at different pH values as indicated (left) and quantitative analysis (right). Nuclei are indicated by dashed lines. Error bars denote s.e.m. (cells: 50). Scale bar: 20 µm. (n=3) **(b)** Effect of S. parasitica growth on the environmental pH in liquid broth. After 3 days of growth (red) the pH decreased from 6.78 to 5 while the control sample (black) shows a constant pH over time. Error bars denote SD (n=3). **(c)** Self-translocation of SpHtp3-mRFP into RTG-2 cells pre-incubated for 1 h with the inhibitor genistein (inhibition of tyrosine kinases) at pH 5.5. Nuclei are indicated by dashed lines. Error bars denote s.e.m. (cells: 50). *** $p < 0.001$ (t-test). Pictures were taken with a Zeiss Imager M2. Scale bar: 20 µm. (n=3) **(d)** Autonomous translocation activity of SpHtp3-mRFP into living RTG-2 cells pre-incubated for 48 h with the sulfation inhibitor $NaClO_3$ (70 mM) at pH 5.5. Scale bar: 20 µm. (n=3)

Supplementary Table 1. SpHtp3-like nucleases from other pathogenic fungi and oomycetes

accession number	species	pathogenicity	signal	prediction	Snase fold
770304069	Aspergillus parasiticus	human	anchor	0.56	7.30E-21
662522722	Aureobasidium pullulans	plants	anchor	0.641	9.40E-25
701776548	Beauveria bassiana	athropods	anchor	0.596	2.20E-25
646295409	Botryobasidium botryosum	wood decay	secretion	0.938	6.00E-23
477537143	Colletotrichum orbiculare	melon, cucumber	anchor	0.843	1.30E-21
663447725	Cyberlindnera fabianii	human	secretion	0.661	3.40E-20
45198453	Eremothecium gossypii	cotton	secretion	0.626	2.60E-22
800921033	Hanseniaspora uvarum	human	anchor	0.628	8.90E-25
799244733	Hirsutella minnesotensis	soybean	anchor	0.939	1.20E-19
574143861	Kluyveromyces marxianus	human	anchor	0.596	8.20E-26
396472204	Leptosphaeria maculans	crops	anchor	0.817	4.90E-25
389632065	Magnaporthe oryzae	plants	anchor	0.652	2.10E-24
835895603	Magnaporthiopsis poae	grass	anchor	0.751	1.30E-21
597572499	Marssonina brunnea	plants	anchor	0.777	4.90E-26
734659414	Metarhizium album	plants	anchor	0.789	1.60E-24
634347841	Microbotryum violaceum	plants	anchor	0.733	5.30E-24
74644366	Nakaseomyces delphensis	human (?)	secretion	0.514	4.40E-24
530542102	Nosema apis	bees	anchor	0.992	8.20E-25
927380789	Ogataea parapolymorpha	insect	secretion	0.605	4.70E-24
531866348	Ophiocordyceps sinensis	moths larvae	anchor	0.743	1.80E-23
859270412	Penicillium brasilianum	onion	anchor	0.839	1.20E-20
599391454	Phanerochaete carnosa	plane trees	secretion	0.982	1.80E-14
695456669	Phytophthora sojae	soybean	anchor	0.985	6.40E-23
695113319	Pichia kudriavzevii	human	secretion	0.951	5.30E-25
750968359	Pisolithus microcarpus	ectomycorrhizum	secretion	0.813	7.60E-11
430814188	Pneumocystis jirovecii	human	anchor	0.778	2.10E-24
913866265	Puccinia sorghi	maize	secretion	0.772	1.20E-18
189203167	Pyrenophora tritici-repentis	plants	anchor	0.939	1.90E-24
751838924	Rhizoctonia solani	plants	anchor	0.69	2.40E-16
472582507	Rhodosporidium toruloides	bovine	anchor	0.758	5.70E-24
813128551	Saprolegnia parasitica	fish	secretion	0.737	7.90E-23
666868197	Scedosporium apiospermum	human	anchor	0.797	1.10E-23
453082712	Sphaerulina musiva	poplar trees	anchor	0.649	8.80E-21
523777553	Spraguea lophii	fish	anchor	0.926	1.30E-19
367042838	Thielavia terrestris	human	anchor	0.666	2.70E-22
367016471	Torulaspora delbrueckii	human	secretion	0.649	4.10E-24
440493309	Trachipleistophora hominis	human	secretion	0.999	1.10E-25
296421569	Tuber melanosporum	ectomhyrizium	anchor	0.501	7.90E-19
632915299	Ustilaginoidea virens	rice	anchor	0.926	6.50E-24
667635355	Vavraia culicis	mosquito	secretion	0.889	9.90E-26
685941421	Wallemia ichthyophaga	human	anchor	0.593	4.90E-24
796695363	Zymoseptoria brevis	grass	anchor	0.695	2.80E-21

Prediction of a secretion signal or membrane anchor for secretion was performed with SignalP 2.0 and prediction of a SNase fold for a dual nuclease function was done with Pfam-A domain search.

Supplementary Table 2. Analysis of the additional band from SDS-PAGE after crosslink

Accession	Description	Score	Coverage	# Unique Peptides	# Peptides	# PSMs	# AAs	MW [kDa]
SPRG_03573T0	SpHtp3	1550.47	70.62	22	22	65	211	23.8
SPRG_04986T0	SpHtp1	135.09	24.00	4	4	8	200	21.6
SPRG_07885T0	histone H4	28.19	21.78	2	2	2	101	11.3
SPRG_15039T0	unknown	27.13	1.80	1	1	1	724	79.7
SPRG_14283T0	unknown	23.68	1.60	1	1	1	501	55.8
SPRG_04290T0	unknown	20.96	0.66	1	1	1	1213	133.3

SpHtp1-His_6 and SpHtp3-His_6 were co-incubated and cross linked with 4 % PFA/PBS (Fig. 6). An additional band appeared which contained peptides for both proteins according to LC-MS/MS analysis.

Fig. 2b

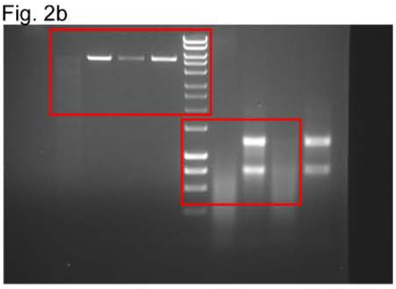

Fig. 4c

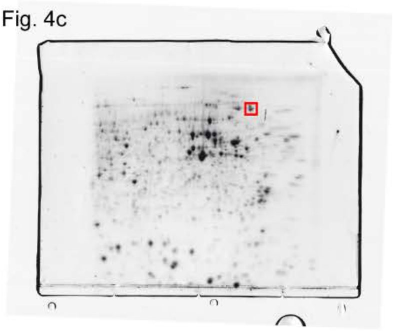

Fig. 4c

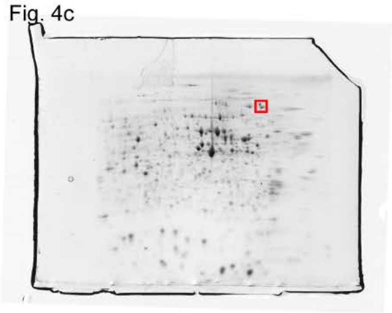

Fig. 4c

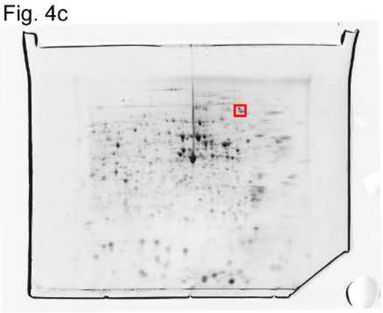

Fig. 4d

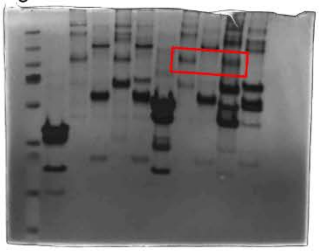

Fig. 4e

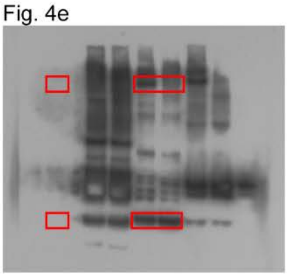

Fig. 4f

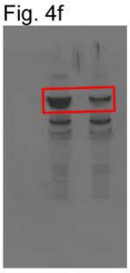

Fig. 4f

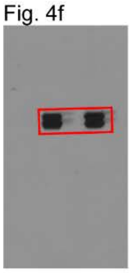

Fig. 6f

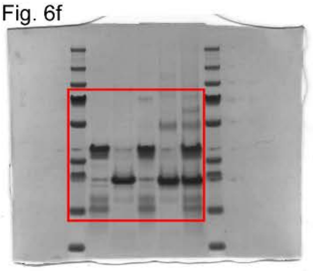

Fig. S1c

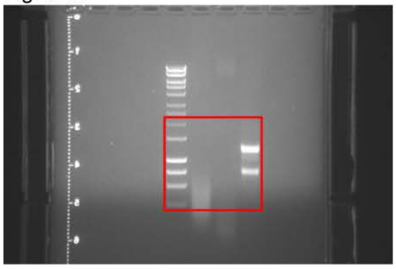

Fig. S1d

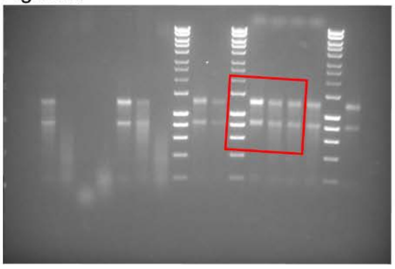

Fig. S1e

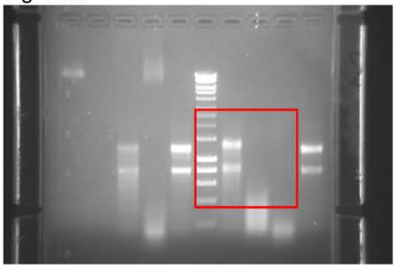

Originals
