# Supplementary material for: Cell entry of a host-targeting protein of oomycetes requires gp96
Source: Nat Commun. 2018 Jun 14;9:2347. doi: 10.1038/s41467-018-04796-3 (PMC6002402; doi:10.1038/s41467-018-04796-3)
Supplement: Supplementary file 2 — Description of Additional Supplementary Files [file 41467_2018_4796_MOESM2_ESM.pdf]

Description of Additional Supplementary Files

File Name: Supplementary Movie 1

Description: RTG-2 cells were incubated at room temperature for 1 h with 3 µM SpHtp3$^{21-211}$mRFP(His)$_6$ at pH 5.5. Subsequently, cells were washed extensively before challenging with *S. parasitica* cysts and a 3 h incubation. mRFP fluorescence and DIC were recorded for ~70 minutes by confocal microscopy. In total 70 frames were taken, each comprising a stack of 10 optical slices (zseries) to cover vesicles which would move to a different focal plane. A 3D(Z)-projection (sum of all 10 optical slices) of the mRFP fluorescence was made per frame. The corresponding movie is shown. The DIC channel corresponds to optical slice 6. SpHtp3$^{21-211}$mRFP(His)$_6$ can clearly be detected within vesicles inside the cells and a reduction in red fluorescence/red vesicles can be observed in a cell being under attack by S. parasitica (see also Fig. 7b and c).
